# Targeting aged bone marrow for systemic rejuvenation

**DOI:** 10.18632/aging.102838

**Published:** 2020-02-06

**Authors:** Faisal J. Alibhai, Ren-Ke Li

**Affiliations:** 1 Toronto General Hospital Research Institute, Toronto General Hospital, Toronto, ON, Canada; 2 Division of Cardiac Surgery, Peter Munk Cardiac Centre, Toronto General Hospital and University of Toronto, Toronto, ON, Canada

**Keywords:** aging, bone marrow, rejuvenation, myocardial infarction, stem cells, inflammation

Aging is a major risk factor for chronic diseases and is directly linked to increasing mortality and healthcare costs worldwide. A key contributing factor to the aging process is a loss of stem/progenitor cell function which leads to a dysregulated tissue microenvironment and reduced repair/regeneration [1]. Over the last two decades scientists have illustrated the importance of intrinsic stem cells for maintaining tissue function and that the number/function of stem cells in a tissue correlates with the repair capacity. One approach that has been taken to increase the number of stem cells in aged tissues is cell transplantation. However, this approach is insufficient due to the poor ability of transplanted cells to survive in recipient tissues. Given that the maintenance of stem cells is crucial for preserving tissue function with aging, identifying approaches which rejuvenate stem cell numbers/function in aged tissues may limit the adverse effects of aging and improve tissue repair.

The bone marrow is an important reservoir of stem/progenitor cells which cross-talk with peripheral organs to help maintain tissue function. Hematopoietic stem/progenitor cells (HSCs) are responsible for producing blood cells throughout life and these downstream cells play an active role in maintaining tissue homeostasis. With aging reduced function of bone marrow cells correlates with dysfunction of peripheral organs. For example, the decline in immune function with age, referred to as immuno-senescence, contributes to the accumulation of senescent cells, persistent low grade inflammation, and reduced responses to injury [2]. The bone marrow is also an important source of endothelial progenitor cells (EPCs) which participate in the generation and repair of vasculature endothelium; aging leads to a decline in circulating EPC number and function. Different strategies have been proposed to rejuvenate the aged bone marrow such as pharmacological treatments, gene therapy, and dietary interventions. However, most approaches have focused on the effect of rejuvenation on HSC differentiation and EPC colony formation rather than effects on peripheral tissues. Therefore, we hypothesized that reconstituting aged mice with young bone marrow leads to stable engraftment of young cells in aged mice and rejuvenates tissue repair responses [3].

We recently utilized this bone marrow rejuvenation approach to study the effect aging has on the repair processes initiated post-myocardial infarction [4]. Aged mice were reconstituted with young Sca-1^+^ bone marrow stem cells and examined 4 months later to allow cross talk between the bone marrow and heart. Young bone marrow reconstitution rejuvenated cardiac endothelial cells which contributed to improved repair and better outcome following myocardial infarction. Mechanistically, young cells which migrated to the heart expressed more *Cxcl12* and *Vegf*, key cytokines involved in angiogenic responses. This correlated with greater expression of CXCR4 and phospho-AKT in resident cardiac endothelial cells isolated from mice receiving young vs. old bone marrow. In addition to improved angiogenesis, our lab has shown that rejuvenation using reconstitution of young cells improves multiple repair processes. Young bone marrow cell transplantation increases the proliferation of resident cardiac cells [3], increases epicardial derived cell migration/activation [5], and enhances the acute inflammatory response following myocardial infarction in aged mice [6]. Together these changes act to improve infarct healing, limit remodeling, and slow the progression to heart failure. These studies highlight the potential benefits of bone marrow rejuvenation on tissue repair in aged individuals.

Beyond cardiac repair, we have shown that bone marrow cells interact with other tissues and that bone marrow rejuvenation can benefit multiple organ systems. Reconstituting aged mice with young cells leads to the repopulation of the retina with young bone marrow derived microglia [7]. Within the retina these cells secrete cytoprotective factors such as fibroblast growth factor-2 and insulin-like growth factor-1 which limit cell death following ischemia/reperfusion injury. More recently, we also demonstrated that bone marrow rejuvenation leads to the introduction of young bone marrow-derived microglia in the brain and that these cells act to improve learning and memory responses compared with mice receiving old bone marrow [8]. Mechanistically, young bone marrow-derived microglia adopt a neutral or anti-inflammatory phenotype while old bone marrow-derived microglia adopt a pro-inflammatory phenotype. These results are consistent with studies which have linked increased neuro-inflammation to a decline in cognitive function.

**Figure 1:**
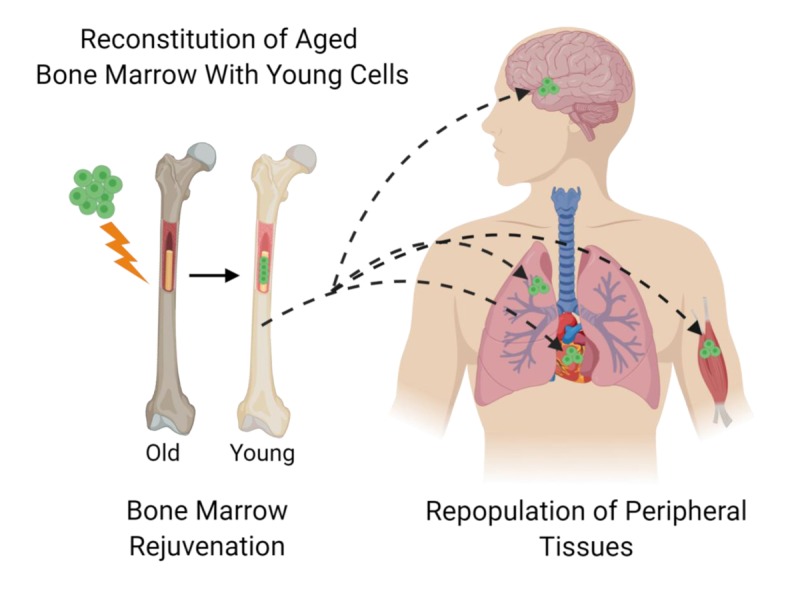
**Systemic rejuvenation using young bone marrow.** Schematic diagram showing that following reconstitution of old bone marrow with young cells the transplanted cells can repopulate peripheral organs such as the heart, lung, brain, and skeletal muscle. Young bone marrow cells interact with host cells to influence cellular function and act to improve tissue responses to injury.

Together our studies as well as studies by others demonstrate that introducing young bone marrow into aged mice is an effective rejuvenation strategy with systemic benefits (Figure 1). Bone marrow-derived cells are in constant communication with peripheral tissues and progressively populate these tissues with aging. Transplanting young bone marrow cells into aged animals introduces a constant source of young healthy cells that act to maintain tissue function. Although this rejuvenation approach is effective, the methods used to transplant young cells have side effects as they require irradiation or chemotherapy. Future rejuvenation approaches which target the stem cell microenvironment comprising of bone marrow niche and stem/progenitor cells may promote in-situ rejuvenation without the need for transplant procedures. Moreover, understanding the effect bone marrow rejuvenation has on the host environment will be essential as bone marrow transplant only replaces dysfunctional aged cells with functional young cells. Co-treatment with therapies which target terminally differentiated cells such as cardiomyocytes and neurons may help to further restore aged tissue function. Despite these limitations, bone marrow transplant studies have been pivotal in demonstrating that the decline in bone marrow function contributes to impaired tissue repair and that bone marrow rejuvenation can have systemic benefits. Rejuvenation of the aged bone marrow is an exciting approach aimed at improving the healthspan and lifespan of aged individuals.
